# Medical device related pressure ulcers in Jordan: Prevalence study among critically ill patients

**DOI:** 10.1002/hsr2.620

**Published:** 2022-05-05

**Authors:** Yahya W. Najjar, Mohammad Y. Saleh, Zeinab M. Hassan

**Affiliations:** ^1^ Zarqa University College Al‐Balqa Applied University Zarqa Jordan; ^2^ School of Nursing University of Jordan Amman Jordan; ^3^ School of Nursing The Hashemite University Zarqa Jordan

**Keywords:** Jordan, medical device, pressure ulcer, prevalence

## Abstract

**Background:**

Medical device‐related pressure ulcers are increasingly common in critical care units. These ulcers can be complicated due to the necessity of the device for diagnosis or treatment.

**Purpose:**

To determine the prevalence of and risk for medical device‐related pressure ulcers in critical care units in Jordan in addition to identifying the preventive measures for those ulcers as well as identifying the most frequently used medical devices that cause ulcers and to assess the relationships between prevention measures and developing ulcers.

**Methods:**

A cross‐sectional survey was used to assess ulcers among 318 patients who were elder than 18 years old. Data collection was based on an outline published by the European Pressure Ulcer Advisory Panel, Braden Scale, and an author‐developed specific checklist.

**Results:**

The prevalence rate of medical device‐related pressure ulcers was 38.1%. Most affected sites were sacrum and heel, and most affected were those with old age, being admitted to public hospitals, and with a prolonged hospital stay. About half of the patients (46.3%) had severe risk. Only 17% of the patients who were at risk got adequate preventive measures. Face masks, endotracheal tubes, pulse oximetry probes, and intravenous catheters were associated with almost half of the ulcers.

**Conclusion:**

Medical device‐related pressure ulcers are threats to patient safety and quality of nursing care in hospitals, which require determining appropriate preventive measures. Key messages:

Medical device‐related pressure ulcers are common among patients in critical care units, which raise the need to evaluate the prevalence of such type of ulcers in those patients.

Three hundred and eighteen patients were investigated for the prevalence of medical device‐related pressure ulcers through a cross‐sectional survey.

Patients in critical care units in Jordan had a high prevalence rate for medical device‐related pressure ulcers, which require the need to apply appropriate preventive measures.

## 1 INTRODUCTION

The employment of advanced technology is essential to provide care for hospitalized patients, especially in critical care units (CCUs). Using medical devices (MDs) in physically compromised critically ill adults, children and trauma/orthopedic patients are pervasive in sustaining life and promoting healing during patients' hospital stay.^1^, ^2^ With the MDs used, however, the risk of an MDs‐related pressure ulcer (MDRPrUs) or “pressure injury” is inevitable.^3^ For instance, critically ill patients who are on a sustained life support system are 2.4 times more probable to acquire hospital‐acquired pressure ulcers (HAPUs) when being compared to patients without a device during their hospital stay.^1^, ^4^, ^5^ The MDRPrUs were defined as “the injury that results from any MD used for diagnosis or treatment and this injury could be fit in the shape of the MD”.^6^

Pressure ulcers (PrUs) are the main health alteration, which results in decreased value of life and increased demands on healthcare systems globally. However, PrUs are largely preventable when available clinical guidelines available are applied by clinicians.^7^ While the prevalence and prevention of common types of PrUs in adult patients have been extensively explored.^8^ The correlation of MDs with acquiring PrUs in hospitalized patients is not well defined.^9^ The National Pressure Ulcer Advisory Panel (NPUAP), however, has set studying MDRPrUs as a priority.^10^

Few studies have captured information on MDRPrUs among critically ill patients. Coyer et al.^11^ conducted a prospective study and assessed the prevalence, severity, sites, etiology, treatment, and healing of MDRPrUs in critical care patients in Australia and the USA. Coyer et al.^11^ reported that 3.1% had MDRPrUs with an average age of 60.5 years, and most of them were men and overweight. Endotracheal tubes (ET) and nasogastric tubes (NGT) were the reason for most MDRPrUs, and repositioning (changing MD position to relieve pressure on the ulcer) was the most common treatment for MDRPrUs (not changing MD position can exacerbate MDRPrUs). Similar to our study's methods and population, a recent point prevalence cross‐sectional study found that the prevalence of MDRPrUs was 19.2% in 146 critically ill adult patients from India.^12^

The MDRPrUs were mostly caused by noninvasive ventilation masks (NIV) and NGT and were associated with a longer stay in intensive care units (ICU). In a closer figure to the Jordanian population, Amirah et al.^13^ studied 431 adult patients receiving intensive care in a huge acute care hospital in the kingdom of Saudi Arabia and found that 115 patients (26.7%) had a minimum 1 MDRPrUs. The overall number of all PrUs was 395; 128 of these were MDs related (32.4%).

Other researchers reported that the percentage of PUs could reach up to 49% of the ICU patients^14^ and the majority (68%) occurring in intensive care.^15^ Hence, under‐reporting of MDRPrUs is evident because nearly 74% of them are not clinically recognized till they are unstageable, stage III, or stage IV, and there has been poor documentation of device removal, and pressure relief, and/or skin inspections. Most of the studies revealed that stage II is considered the most common MDRPrUs.^1^, ^16^ PrUs are categorized based on NPUAP and the European Pressure Ulcer Advisory Panel (EPUAP) Classification System into six stages: unstageable, suspected deep tissue injury, stages I–IV.^6^

It is challenging to compare the prevalence and incidence of MDRPrUs due to variations in the population under investigation, the location, and stage of ulcers, and the MDs used. For instance, 35% of MDRPrUs developed on the ears^1^ in an adult population, and nearly 50% were developed on the nose and the feet of neonates and children. The overall prevalence of MDRPrUs has been reported globally with variable values; for example, Amirah et al.^13^ found that the prevalence of MDRPrUs in Saudi Arabia was 29.7%. However, Coyer et al.^11^ reported in their study in the USA and Australia that the prevalence rate of MDRPrUs was 4.1%. On the contrary, Kayser et al.^17^ found in their study in the USA and Canada that the prevalence rate of MDRPrUs was 0.6%.

Regarding the devices that cause the MDRPrUs, ET tube and Foley's catheters,^13^ cervical collar or braces,^2^ oxygen therapy tubes and masks,^12^ and feeding tubes such as nasogastric and jejunal tubes were reported as the most common devices that cause MDRPrUs.^12^ The following factors can increase the possibility and risk of developing MDRPrUs: hard device material, improper choice of MD, attaching MD on an area with little fat tissue, moisture condition of the skin where the device is attached, improper fixation method, improper use of adhesive tape, using many types of MDs, and using MD on the same area for a long period.^18^ In general, old age and male gender, prolonged hospital stay, and having PrUs in the body are major risk factors that increase the possibility of developing MDRPrUs.^19^

In the Middle East, evidence has revealed that common types of PrUs lack adequate documentation, risk assessment, training, prevention, and treatment guidelines for nurses.^20^ Only one published work on the prevalence of MDRPrUs among critically ill patients has been found in the Arab world, providing prevalence numbers from the Kingdom of Saudi Arabia.^13^ The purpose of this study is to describe the prevalence of and the most communal MDs causing MDRPrUs in patients who are critically ill in Jordan, in addition to assessing the risk level for developing MDRPrUs and the preventive measures for MDRPrUs.

## 2 METHODS

### 2.1 Study design

A cross‐sectional survey design was used to describe the prevalence of MDRPrUs and common MDs causing MDRPrUs in critically ill patients in Jordan.

### 2.2 Sample and setting

Hospitals having CCUs, and a capacity of 200 beds or more were surveyed as such hospitals contained more patients who were vulnerable to acquiring MDRPrUs in their CCUs. Of the 50 invited hospitals, 11 met the inclusion criteria and 10 gave permission to access critically ill adult patients including three public hospitals (816 beds) (9 CCUs comprised 100 beds), four private (794 beds) (10 CCUs comprised 130 beds), one‐non‐profit (442 beds) (two CCUs comprised 30 beds), and two university hospitals (1099 beds) (nine CCUs comprised 100 beds). The occupancy rate of CCUs in Jordan is almost 100%.

### 2.3 Ethical considerations

Ethical authorization was obtained from the institutional review board committee in the university where the principal researcher works; additional approvals were obtained from ethical research committees in the participating hospitals. Each patient or his first‐degree relative (if the patient's level of consciousness was 8 or less according to the Glasgow coma scale assessment by the caring team) was asked to sign a consent form for participation in the survey. The patient's right to withdraw from the research study at any stage was guaranteed.

### 2.4 Data collection

A list of patients was obtained from the Directors of Nursing of each targeted hospital on the assigned day for data collection. A critically ill patient is defined as a patient with severe respiratory, cardiovascular, or neurological alterations, often in combination, and are reflected in abnormal physiological observations.^21^ All patients at selected CCUs were assessed for eligibility criteria and those patients who conformed to the following were included in the study; aged 18 years and above, admitted before midnight on the predefined day of data collection, using MD at the time of the study, having pressure ulcer (PU) stages I–IV according to the NPUAP, EPUAP classification system^6^ as assessed by the nursing staff caring for them and the researcher, and patients with a Braden score of ≤18.

Each patient was assessed by a wound care nurse assigned in each hospital selected, so 10 nurses assisted in the data collection procedure. Those nurses were trained about how to use the data collection measures in assessing the target patients (i.e.: how to calculate prevalence, risk assessment, how to use the Braden scale, in addition to orientation about MDs that could cause PrUs). The training program lasted for 2 days, then each nurse was asked to use the data collection measures clinically on two patients (not from the original sample) therefore the pilot study sample was 20 patients. These nurses displayed almost 0.90 interclass correlation coefficient in grading results of risk assessment and Braden scale and were consistent with the assessment done by one of this study's investigators who is a tissue viability nurse specialist.

Observations for patients selected for the study by the assigned nurses to collect data were managed in unscheduled visits through the day and night shifts. Assessment of patient skin was carefully performed and each patient was assessed for PrUs on daily basis during the data collection period identified below. PrUs from stage I (nonblanchable erythema) to stage IV (widespread destruction of the skin and underlying tissue), deep‐tissue injury, and unstageable ulcers were identified according to the NPUAP, EPUAP classification system.^6^ Risk assessment and the use of preventive measures were documented. After completing data collection, data sheets were checked and any absent data were added. The data were collected between August and November 2019.

### 2.5 Measures

Data were collected using a modified form based on the EPUAP pressure ulcer prevalence survey form, which has been validated and showed high interrater reliability.^22^ The initial form of the survey was subjected to a validation process and pilot testing by the researchers and a group of expert wound care nurses (*n* = 10) from a university teaching hospital. This improved the content validity of the form, the level of comprehensiveness, clarity, and reduced ambiguity. The modified survey form included data across five categories: general patient data, risk assessment (The Braden score), MDs used, skin inspection, and utility of pressure ulcer prevention interventions. The first category describes the patients' age, gender, duration of stay (from admission until the survey time), previous hospitalization, and the patient's diagnostic category. It also includes details of the hospital including sector, the total number of beds, and the unit's number of beds. The second category includes patients' assessment using the Braden scale to identify their risk of PU development. Risk assessment of PU development using the Braden scale is based on six subscales (Sensory Perception, Activity, Mobility, Moisture, Nutrition, and Friction/Shear) and scores on each of these six subscales range from 1 to 4 except for friction/shear which ranges from 1 to 3; lower scores indicate a high risk for PU development. Scores of 15 to 18 indicate mild risk, scores of 13–14 indicate moderate risk, scores of 10–12 indicate high risk, and scores of 9 or less indicate very high risk.^23^

The third category includes a checklist developed to observe and record (MDRPrUs). The checklist included 26 MDs that are associated with PU development and were informed by literature, as follows: antiembolic stockings;^1^ cervical collars;^24^ ET tubes/commercial ET tube holders; face masks for noninvasive positive pressure ventilation;^25^ facial containment devices;^26^ nasal cannulas, sequential compression devices, chest tubes/drains of any type, electrocardiogram (ECG) leads;^27^ pulse oximetry probes;^1^ radial artery catheters;^1^ splints and braces, traction devices;^28^ urinary catheters;^29^ a cast for clubfoot correction;^30^ electroencephalogram (EEG) leads, epidural catheters/nerve blocks, hubs/tubings; enteral feeding tubes (any type);^31^ intravenous catheters;^26^ NIV equipment;^32^ tracheostomy tubes;^26^ and wheelchairs.^33^ The fourth category includes clinical assessment of the patient's skin. All patients at risk for PU (Braden score ≤ 18) were examined for the presence of PU, PU stages, and PU location using the NPUAP, EPUAP classification system.^34^ The last category of the survey is the prevention measures used as recorded by the caring team. Prevention included skin inspection, handling of the patient's body, supporting MD (defined as padded skin before device application and/or no support used), use of PU prevention guidelines, using skin barrier creams, nutritional screening, and nutritional supplements, MD repositioning as well as patient repositioning which are documented as planned frequently, or not planned/irregular.

### 2.6 Data analysis

Descriptive analysis of data was accomplished using IBM SPSS 24.0.^35^ Preliminary data screening was conducted to identify any missing data. Descriptive statistics including frequencies were used to describe study variables. All percentages were rounded to the nearest digit. The prevalence of MDRPrUs was calculated based on the number of patients who developed MDRPrUs and the total number of patients included in the sample.

## 3 RESULTS

The number of patients congruent with the inclusion criteria in the selected hospitals was 348 critically ill patients, 13 patients dropped and 17 patients were not obtainable at the time of screening to give a final sample of 318 patients. The demographic characteristics are shown in Table 1. Only hospital sector, age, and length of patient stay in hospital were significantly correlated with the occurrence of MDRPrUs, that is increasing age and prolonged length of stay in hospital as well as being admitted to public hospitals increased the risk for MDRPrUs.

**Table 1:** The demographic characteristics of the patients (*N* = 318).

Patient's characteristics		*n* (%)	With MDRPrUs *n* = 121	Without MDRPrUs *n* = 197	*p*‐value
Hospital sector	Public	79 (24.8%)	43 (35.5%)	36 (18.3%)	0.008^1*^
	Private	127 (39.9%)	37 (30.6%)	90 (45.7%)	
	Nonprofit	26 (8.2%)	8 (6.6%)	18 (9.2%)	
	University	86 (27.0%)	33 (27.3%)	53 (26.9%)	
Gender	Male	197 (61.9%)	67 (55.4%)	130 (66%)	0.086^1^
	Female	121 (38.1%)	53 (43.8%)	67 (34%)	
Age (in years)			60.5 (18.6)	53.3 (17.9)	0.001^2^
*M (SD)*			*R* = 18‐90	*R* = 18‐100	
Length of stay (days)			13.08 (18.4)	4.2 (4.4)	<0.001^2*^
*M (SD)*			*R* = 1–90	*R* = 1–37	
Previous hospitalization	Yes	266 (83.6%)	105 (86.8%)	161 (81.7%)	0.176^1^
	No	52 (16.4%)	15 (12.4%)	36 (18.4%)	
Diagnosis	Medical	190 (59.7%	81 (66.9%)	109 (55.3%)	0.095^1^
	Surgical	97 (30.5%)	32 (26.4%)	65 (33%)	
	Oncology	31 (9.7%)	8 (6.6%)	23 (11.7%)	

*Note*: M: mean, SD: standard deviation, R: range [minimum–maximum]). *Statistically significant at α = 0.05 using Chi‐squared test (1) and Mann–Whitney test (2).

### 3.1 Prevalence of MDRPrUs

Table 2 shows that 121 (38.1%) critically ill adult patients had at least one or more (MDRPrUs) stages I to IV, totaling 132 ulcers. Of these, 102 (77.3%) PrUS were stages I and 30 (22.7%) were in stage II. The most communal anatomic sites for MDRPrUs were hands and arms (*n* = 39, 29.5%), lips (*n* = 20, 15.1%), and cheeks (*n* = 16, 12.1%). Excluding stage I PU, the prevalence rate was 8.4% (*n* = 27), a higher rate of MDRPrUs was recorded among critically ill adult patients from public (*n* = 43, 35.5%), private (*n* = 37, 30.6%), and university (*n* = 33, 27.3%) compared with lower rate in nonprofit‐hospital (*n* = 8, 6.6%) (Table 1).

**Table 2:** Description of medical device related pressure ulcers (MDRPrUs) among critically ill adult patients.

Characteristics of MDRPrUs		*n* (%)
Prevalence (*N *= 318)	Including stage 1 Excluding stage 1	121 (38.1%) 27 (8.4%)
Number of ulcers among patients with MDRPrUs (*n* = 121)	One ulcer	110 (90.9%)
	Two ulcers	11 (9.1%)
Total number of ulcers		132
Location of ulcers (*n *= 132)	Cheeks	16 (12.1%)
	Neck	11 (8.3%)
	Hands/Arms	39 (29.5%)
	Legs/Feet	9 (6.8%)
	Perineal/Meatal orifice	10 (7.8%)
	Lips	20 (15.1%)
	Chest	5 (3.8%)
	Ears	8 (6.0%)
	Abdomen	7 (5.3%)
	Nose	7 (5.3%)
Stages of ulcers (*n* = 132)	Stage 1	102 (77.3%)
	Stage 2	30 (22.7%)
	Stage 3	0 (0.0%)
	Stage 4	0 (0.0%)
	Unstageable	0 (0.0%)
	Suspected deep‐tissue injury	0 (0.0%)

### 3.2 Risk assessment

All patients involved in this study were at risk of PrUs using the Braden scale. While 197 patients of the study sample were free of MDRPrUs, 55.3% (*n* = 109) of them were evaluated as having a mild risk of PU while the remaining were considered vulnerable to PrUs at different levels including severe, high, and moderate risk. On the other hand, of the patients who had (MDRPrUs) (*n* = 121), 56 (46.3%) patients had severe risk, 24 (19.8%) high risk, 13 (10.7%) moderate risk, and 28 (23.1%) mild risk (see Table 3). Our study results showed a statistically significant (*p* < 0.001) relationship between the Braden scores and the MDRPU development.

**Table 3:** Description of the Braden scores among surveyed patients.

Level of PrUs risk	Overall Braden scores (*n* = 318)	Braden scores among patients had MDRPrUs (*n* = 121)	Braden scores among patients free of MDRPrUs (*n* = 197)
≤9 (pevere risk)	81 (25.5%)	56 (46.3%)	25 (12.7%)
10–12 (high risk)	51 (16.0%)	24 (19.8%)	27 (13.7%)
13–14 (moderate risk)	49 (15.4%)	13 (10.7%)	36 (18.3%)
15–18 (mild risk)	137 (43.1%)	28 (23.1%)	109 (55.3%)

### 3.3 PrUs prevention in patients who had MDRPrUs

Table 4 shows the allocation of PrUs prevention strategies used with patients who had MDRPrUs. Although results revealed adequate manual handling and repositioning of patients having MDRPrUs, an ineffective PrUs prevention was implemented in relation to both nutritional screening and padding skin before device application (*n* = 34, 28%). Although patients who had MDRPrUs were evaluated at risk of PU, no single patient received planned frequent relocation of the MD since implemented. Other components of the prevention program were relatively implemented such as the use of PU clinical guidelines (*n* = 76, 62.8%), frequent skin inspection (*n* = 81, 66.9%), and using skin barrier creams (*n* = 68, 56.2%). The results in Table 4 demonstrated that none of the correlations were statistically significant at *α* = 0.05 using the chi‐squared test. As all prevention and treatment variables were not significant, performing regression analysis to elucidate a prediction model among those who developed MDRPUs was useless and therefore the study results were dependent mainly on correlations.

**Table 4:** Allocation of pressure ulcer (PrUs) prevention measures used in patients who had medical device‐related pressure ulcers (*N* = 121).

Prevention measures	*n* (%)	*p‐*value
Use of PrUs prevention guidelines		0.197
Yes	76 (62.8%)	
No	45 (37.2%)	
Skin inspection		0.475
Yes	81 (66.9%)	
No	40 (33.1%)	
Skin barrier creams		0.371
Yes	68 (56.2%)	
No	53 (43.8%)	
Handling of the patient's body		0.897
Manual handling	120 (99.1%)	
Mechanical handling	1 (0.9%)	
Repositioning of the body		0.927
Planned frequently	120 (99.1%)	
Not planned/irregular	1 (0.9%)	
Nutritional screening		0.533
Yes	34 (28.0)	
No	87 (72.0%)	
Nutritional supplements		0.459
Yes	43 (35.5%)	
No	78 (64.5%)	
Support medical devices		0.927
Pad skin before device	34 (28.0)	
Application	87 (72.0%)	
No support used		
Repositioning of the medical		0.113
Device	11 (0.09%)	
Planned frequently	111 (91%)	
Not planned/irregular		

### 3.4 Medical device‐related pressure ulcers (MDRPrUs)

Our study indicated the use of 26 MDs in CCUs in Jordan. The frequently used MDs are shown in Table 5. Results revealed that face masks for noninvasive positive pressure ventilation, ET tubes (ET tube and holders), pulse oximetry probes, and intravenous catheters were associated with almost half of MDRPrUs in CCUs in Jordan. Although ECG leads (*n* = 242), noninvasive blood pressure amplifier (*n* = 240), urinary catheters (*n* = 233), cardiac monitoring cables and temporary pacemakers (*n* = 197), and patient identification bands (*n* = 179) were frequently used in critically ill patients, they were poorly associated with MDRPrUs.

**Table 5:** The frequently used and rank order of medical devices causing medical device related pressure ulcer (MDRPrUs).

Rank order	Medical devices	Frequency of use *n* (%)^a^	Frequency of medical devices caused MDRPrUs*n* (%)
1	Face mask for noninvasive positive pressure ventilation	75 (23.5%)	21 (15.9%)
2	Endotracheal tubes/commercial endotracheal tube holders	102 (32%)	19 (14.4%)
3	Pulse oximetry probes	260 (81.7%)	17 (12.9%)
4	Intravenous catheters: hubs/tubing	206 (64.8%)	15 (11.4%)
5	Enteral feeding tubes (any type)	63 (19.8%)	11 (8.3%)
6	Urinary catheters	233 (73.3%)	9 (6.8%)
7	Diapers	136 (42.8%)	6 (4.6%)
8	Nasal cannulas	56 (17.6%)	5 (3.8%)
	Patient ID bands	179 (56.2%)	5 (3.8%)
	Tracheostomy tubes	22 (6.9%)	5 (3.8%)
9	Noninvasive blood pressure amplifier	240 (75.5%)	4 (3.0%)
10	Cervical collar	8 (2.5%)	2 (1.5%)
	Condoms	8 (2.5%)	2 (1.5%)
	Chet tubes/drains of any type	12 (3.8%)	2 (1.5%)
11	Restraints	8 (2.5%)	1 (0.75%)
	Bedpan/Urinal	12 (3.8%)	1 (0.75%)
	Anti‐embolic stockings	20 (6.2%)	1 (0.75%)
	Radial artery catheters	22 (6.9%)	1 (0.75%)
	Sequential compression devices	2 (0.6%)	1 (0.75%)
	Traction devices	2 (0.6%)	1 (0.75%)
	Draw sheets	17 (5.3%)	1 (0.75%)
	Noninvasive ventilation equipment	9 (2.8%)	1 (0.75%)
	Electrocardiogram (ECG) leads	242 (76.1%)	1 (0.75%)
12	Temporary pacemaker/cardiac monitoring cables	197 (61.9%)	0 (0.0%)

^a^ Multiple devices reported among the same patients.

## 4 DISCUSSION

The overall prevalence of MDRPrUs was 38.1% and declined to 8.4% when excluding stage one PU. MDRPrUs (stages II–IV) are responsible for one‐third of all PrUs in this sample. Stages I and II MDRPrUs were the most communal; while unstageable PrUs and deep tissue injury were not found. Hence, MDRPrUs is a growing problem and required the need for future research and consensus.

It is hard to compare our data with that of other studies. Only two studies identified MDRPrUs in critically ill patients.^11^, ^13^ Our findings were extremely higher than the 1.3% prevalence stated by Coyer et al.^11^ in the USA and Australia and the 26.7% prevalence stated by Amirah et al.^13^ in Saudi Arabia with a relatively similar number of ICU patients. A plausible explanation for higher prevalence might be the sample which represents only ICU patients with few prevention measures implemented, and inclusion of stage one ulcers. Representation of a broader population might have yielded a lower whole prevalence of MDRPrUs. This would be as low as Kayser et al.^17^ report of only 0.60% MDRPrUs prevalence among long term, hospice, and rehabilitation patients. This also would be lower than the 1.4% stated by Black et al.^1^ in an academic acute care medical center that was restricted to patients without a PU existing on admission. Thus, a larger study population including these patients would be more informative.

Compared to other studies in the USA, Australia, and Saudi Arabia which employed similar methods, Jordan shows similar findings related to male gender, age, and length of stay. Despite younger Jordanian patients, the mean age of patients found with MDRPrUs was almost similar across studies. It was similar (*M* = 60.5) in Jordan and the USA and Australia,^11^ while it was older (*M* = 69.6) in Saudi Arabia.^12^ Our results showed that male patients had a higher risk of developing MDRPrUs (55.4%) which was consistent with Coyer et al.^11^ (67%) and Amirah et al.^13^ who found that male patients had a 2.8 times risk for developing MDRPrUs in Saudi Arabia. The mean length of stay was typical (*M* = 13 days) among patients who had MDRPrUs in Jordan, USA, and Australia.^11^ Unlike previous findings, our results were different in nature of the patient's diagnosis and the risk of PU development. About 67% of Jordanian critically ill patients who had MDRPrUs were having medical illnesses compared to 47% of patients from the USA and Australia and 22.6% from Saudi Arabia. Additionally, the mean scores of the Braden scale were lower among Jordanian patients (*M* = 10.5) compared with patients from USA and Australia (*M* = 14).

In the present study, 77.3% of screened MDRPrUs (*n* = 132) were stage I while serious ulcers of stage III or deeper were not present. The percentage of reported stage I MDRPrUs might reflect the fact that mucosal PrUs were not reported and/or staged in this study as researchers did not include this option to mark mucosal PrUs, however, data collectors may have marked them as stage I PUs. Our findings were consistent with several studies^16^, ^35^, ^36^ that found that most MDRPrUs were either stage I or stage II PrUs. It is essential to consider that these studies included PrUs existing on admission and healthcare facility‐acquired MDRPrUs and represented larger samples from broader care settings, however, it is difficult to compare our results across different studies. Additionally, underreporting of serious MDRPrUs might be a significant clinical problem. For instance, Apold and Rydrych^2^ reported that Serious MDRPrUs (stages III, IV, and unstageable) were not found at hospital admission. Since reporting system was refined in 2009, 255 severe HAPUs were stated in 34 hospitals with 113 other hospitals not stating any severe ulcers. In contrast, only 10% of MDRPrUs (2 of 20) were stage III among data set analyzed by Coyer et al.,^11^ in the USA and Australia and were not present at all among our sample, however, the number of MDRPrUs would be considerably under‐reported if only serious ulcers were taken into consideration. Besides absence of serious ulcers in our sample might be attributed to inclusion of only ICU patients and reporting data that focused on all stages of MDR ulcers rather than patients' clinical findings which impedes calculating prevalence and as a result restricts the comparison among sites and studies.

Apold and Rydrych^2^ studied Minnesota's mandatory state reporting data to determine trends in communal root cause MDRPrUs that was stated through the state and created best practices for MDRPrUs prevention due to the use of cervical collars and respiratory therapy and care equipment. Severe PrUs (stages III, IV, and unstageable) that were not existing at hospital admission were stated. In 2009, the state invited together experts, revised their reporting form to purify their root origin analysis, and involved MDRPrUs. From that time, 255 severe HAPUs were stated by 34 hospitals with 113 other hospitals not stating any severe PrUs.

In our current study, how many patients were screened was not stated, so prevalence could not be computed. Also, because state reporting was necessary only for the severe PrUs, the number of less‐serious PrUs was not involved. On the other hand, only 5% of PrUs (1/20) in our set of data were serious, and so the number of MDRPrUs would be considerably undervalued if only serious PrUs were considered. Furthermore, Apold and Rydrych's report^2^ does not take into account ICU and so it is impossible to compare it with our sample.

Our study data indicate how essential it is to evaluate, determine and commence early prevention for potential MDRPrUs. However, stating CCUs data by PrUs rather than by patients impedes computing the prevalence and as a result, restricts comparison within sites and studies.

Although prevention of MDRPrUs is the main goal, earlier detection of MDRPrUs could result in a better prognosis and lower the financial impact of this disease. This can be done with a routine physical examination by the medical team and taking proper precautions. However, unfortunately, there are no clear guidelines in this regard and a consensus has yet to be reached.

The hands and arms of patients in the study sample were the most frequent site of MDRPrUs (29.5%). On the other hand, ulcers of the lips (15.2%), cheeks (12.1%), and other sites of the head and neck taken collectively make MDRPrUs of the head and neck the most common site. This is consistent with the study done by many researchers. This may be attributed to the number of MDs used in this region, ranging from Nasal cannulas, and NGT to ET tubes and tracheostomy tubes.^12^, ^25^

Forthcoming research is required to discover the relationship between the type of MD and the depth of tissue included in the MDRPrUs; certain MDs might be related to advanced PU numerical stages. The results of this present study are different from ICU studies stated by both Coyer et al.^11^ and Amirah et al.^13^ which found ET tubes or NGT to be the most communal MDs related to PrUs.

In this study, ET tubes and NGT encompassed 7.5% and 5.0% of the MDRPrUs, respectively, among all health care settings. Of the MDRPrUs seized in this study, 28% were reported in CCUs settings, as divergent to the aforesaid studies,^37^ which absolutely studied ICU settings. It is probable that a general population uses different MDs than the CCUs population.

BRADEN risk tool was used to assess the threat of acquiring MDRPrUs in our study. It was found that 46.3%, 19.8%, 10.7%, and 23.1% of MDRPrUs patients had a severe, high, moderate, and mild risk, respectively. Although the BRADEN risk tool was related to the prevalence of MDRPrUs in our study, it is a tool used to evaluate the threat of acquiring PrUs in general and other studies have deemed it unreliable.^1^ Thus, further research is advised to conduct a tool to reliably assess the risk of developing MDRPrUs.

This study showed that 35.5% of the patients with MDRPrUs were treated in public hospitals in comparison with 30.6%, 27.3%, and 6.6% at private, teaching, and non‐profit hospitals, respectively. This difference can be attributed to the different devices, settings, types of patients, and procedures performed in each sector. Different protocols and documentation processes can be employed to minimize this difference present among types of hospitals.

We aim to standardize these protocols to provide the best care across the country. As MDRPrUs are a growing problem, we advise further research in the hope of reaching a consensus on prevention strategies, detection, and treatment of this disease. Although we excluded patients who were younger than the age of 18 years, we cannot deny the high prevalence of MDRPrUs in the younger age group, which was found to be 10% for children.^38^ This encourages us to investigate MDRPrUs in different age groups in the future.

### 4.1 Strengths

The participants in the current study included patients from different centers across Jordan, which adds strength to this study. In addition, the use of the EPUAP grading system facilitated homogenizing the results throughout the study population.

### 4.2 Limitations

The exclusion of certain patients, such as those younger than 18 years, can alter the true prevalence of MDRPrUs in Jordan. In addition, the absence of a specific risk tool for developing MDRPrUs made it difficult to assess properly the risk in our patients. Only adult patients were involved in this study, and additional research is necessary in pediatric patients.

The survey concentrates on acute care health care settings, and as a result, the size of the sample from each setting was fairly small, making it unattainable to study variances in features of MDRPrUs among health care settings. Eventually, the current study was limited to self‐reported data from health care facilities that chose to take part in the MDRPrUs survey. Studies using considerable samples from non‐acute health care settings are required to know if these results can be generalized outside acute care.

## 5 CONCLUSION

This study found that the prevalence of MDRPrUs was high, and male patients were the most vulnerable to MDRPrUs. The most communal anatomic locations for MDRPrUs were the hands/arms, and the most frequently associated MDs were the face‐ mask for noninvasive positive pressure ventilation. Due to the absence of assessment and prevention protocols in hospitals, several cases reported during this study did not receive adequate care. This study found the foremost communal anatomic locations for MDRPrUs were the hands/arms, and therefore the most typically related MDs were masks for noninvasive positive pressure ventilation.

## AUTHOR CONTRIBUTIONS

*Conceptualization*: Mohammad Y. Saleh, Yahya W. Najjar. *Data curation*: Mohammad Y. Saleh. *Formal analysis*: Mohammad Y. Saleh. *Funding acquisition*: Mohammad Y. Saleh. *Investigation*: Zeinab M. Hassan. *Methodology*: Zeinab M. Hassan, Mohammad Y. Saleh. *Project administration*: Zeinab M. Hassan. *Software*: Zeinab M. Hassan, Mohammad Y. Saleh. *Supervision*: Zeinab M. Hassan, Yahya W. Najjar. *Validation*: Yahya W. Najjar. *Writing*—*original draft*: Mohammad Y. Saleh, Zeinab M. Hassan. *Writing*—*reviewing and editing*: Yahya W. Najjar.

## CONFLICTS OF INTEREST

The authors declare no conflicts of interest.

## ETHICS STATEMENT

Ethical approval to conduct the study was obtained from the university in which primary investigators works and from the hospitals selected as settings for the study.

## CLINICAL IMPLICATIONS

Medical device‐related pressure ulcers are a crucial marker of patient safety and nursing quality in providing care to patients. Hence, crucial preventive measures for MDRPrUs are needed in Jordanian hospitals, and a scientific approach to MDRPrUs before and when occurred is nonetheless to be devised in Jordanian hospitals. We encourage further research in this field to raise awareness of this problem among the healthcare team.

## TRANSPARENCY STATEMENT

The lead author affirms that this manuscript is an honest, accurate, and transparent account of the study being reported; that no important aspects of the study have been omitted; and that any discrepancies from the study as planned (and, if relevant, registered) have been explained.

## Data Availability

The authors confirm that the data supporting the findings of this study are available within the article. All authors have read and approved the final version of the manuscript and had full access to all of the data in this study and takes complete responsibility for the integrity of the data and the accuracy of the data analysis.
